# Circular RNA circRPPH1 promotes breast cancer progression via circRPPH1-miR-512-5p-STAT1 axis

**DOI:** 10.1038/s41420-021-00771-y

**Published:** 2021-12-06

**Authors:** Yixiang Huang, Wenfang Zheng, Changle Ji, Xuehui Wang, Yunhe Yu, Xiaochong Deng, Xiqian Zhou, Lin Fang

**Affiliations:** Department of Breast and Thyroid Surgery, Shanghai Tenth People’s Hospital, Tongji University School of Medicine, Shanghai, 200072 China

**Keywords:** Breast cancer, Non-coding RNAs

## Abstract

Breast cancer (BC) is one of the most fatal diseases among women all over the world. Non-coding RNAs including circular RNAs (circRNAs) have been reported to be involved in different aspects during tumorigenesis and progression. In this study, we aimed to explore the biological functions and underlying mechanism of circRPPH1 in BC. Candidate circRNAs were screened in dataset GSE101123 from Gene Expression Omnibus (GEO) database and a differentially expressed circRNA, circRPPH1, was discovered in BC. CircRPPH1 expression was higher in the cancerous tissue compared to paired adjacent tissue. Further in vitro and in vivo experiments indicated that circRPPH1 acted as an oncogene in BC. In addition, circRPPH1 was mainly localized in cytoplasm and played the role of miR-512-5p sponge. By sequestering miR-512-5p from the 3′-UTR of STAT1, circRPPH1 inhibited the suppressive role of miR-512-5p, stabilized STAT1 mRNA in BC and finally affected BC progression. In conclusion, these findings indicated that circRPPH1 acted as an oncogene and regulated BC progression via circRPPH1-miR-512-5p-STAT1 axis, which might provide a potential therapeutic target for BC treatment.

## Introduction

Breast cancer (BC) has become the top killer among malignant tumors in female all over the world [1]. Poor prognosis of BC is mostly related to tumor recurrence and metastasis [2]. Although multiple therapies are widely used in the treatment of BC, its incidence and mortality remain high [3]. Early diagnosis and intervention are crucial in reducing BC-related adverse events [4]. Therefore, it is of great significance to find novel biomarkers and therapeutic targets of BC.

Circular RNA (circRNA), a group of endogenous non-coding RNA, has shown its effect gradually on tumorigenesis, progression, recurrence, metastasis and drug resistance in recent years [5]. The functions and mechanisms are mainly related to the subcellular distributions of circRNAs. It has been reported that circRNAs located in cytoplasm act as translation templates or sponges of microRNAs (miRNAs) and proteins, while those in the nucleus can regulate the expression of genes [6]. For instance, cytoplasmic circFBXW7 encoded a 21 kDa peptide and repressed glioma tumorigenesis [7]. CircSATB2 in cytoplasm positively regulated FSCN1 by acting as a sponge of miR-326 in non-small cell lung cancer [8]. CircNSUN2 formed a circNSUN2/IGF2BP2/HMGA2 complex in the cytoplasm, which facilitated the exportation and stabilization of HMGA2 in colorectal cancer [9]. A group of exon-intron circRNAs in the nucleus enhanced the transcription of their parental genes in cis [10]. In addition, due to their single-stranded, covalently closed circular structures without 5′-caps or 3′-poly(A) tails, circRNAs are more resistant to degradation and more stable than linear transcripts [11]. This property makes it possible for circRNAs to become biomarkers for tumor diagnosis [12].

The signal transducer and activator of transcription (STAT) family, which is consisted of a group of transcription factors, plays an important role in multiple biological processes of cancer [13]. STAT1 is a member of STAT family and it serves as a double-edged sword in tumor progression [14]. Recent studies indicated that STAT1 may act as an oncogene in BC progression. PLSCR1-activated STAT1 promoted cancer stem cell-like properties and inducing basal-like BC progression [15]. STAT1 elevated BC proliferation via facilitating ERα transcription [16]. Considering the oncogenic role of STAT1 in BC, it is vital to find out the upstream regulator of STAT1.

In this study, we identified that an aberrant circRNA, circRPPH1, was upregulated in both BC tissues and cell lines. It exerted oncogenic biological functions in BC via acting as a sponge of miR-512-5p to activate STAT1. These findings may provide inspirations in BC diagnosis and therapy.

## Results

### The characteristics of circRPPH1 in BC cells

**Fig. 1:**
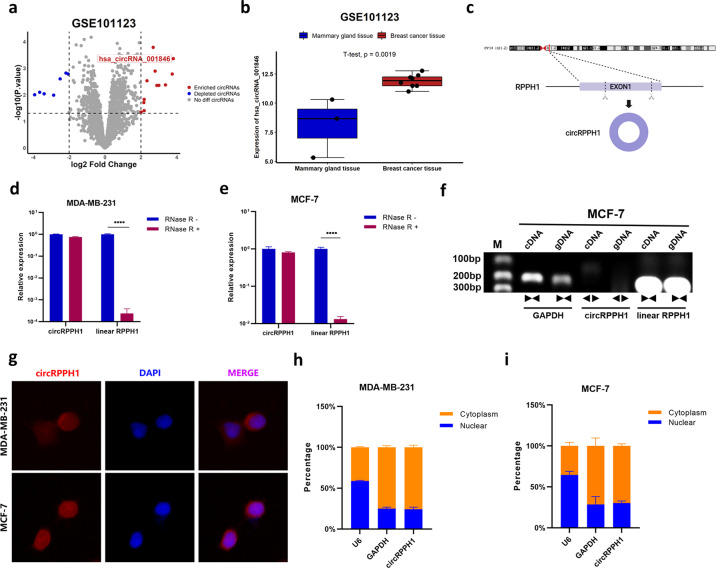
The characteristics of circRPPH1 in BC. **a** Bioinformatics analysis of GSE101123 identified differentially expressed circRNAs including hsa_circRNA_001846 (that is, circRPPH1). The cutoff was set at | log_2_(fold change) | > 2 and *P* < 0.05. **b** The expression of circRPPH1 was analyzed in BC tissue in GSE101123. **c**. CircRPPH1 was formed by backsplicing of RPPH1, exon 1. **d**–**e** The expression of circRPPH1 and linear RPPH1 were detected after treating total RNA of MDA-MB-231 (**d**) and MCF-7 (**e**) cells with RNase R. **f** The circular structure of circRPPH1 was identified by agarose gel electrophoresis. **g** The subcellular distribution of circRPPH1 was detected by FISH assay in MDA-MB-231 (up) and MCF-7 (down) cells. DAPI was used for nucleus staining. Blue, DAPI. Red, circRPPH1. **h**–**i** The levels of U6 (internal reference for nuclear transcripts), GAPDH (internal reference for cytoplasmic transcripts) and circRPPH1 were determined by RT-qPCR after subcellular fraction. *P < 0.05, **P < 0.01, *** *P* < 0.001, **** *P* < 0.0001.

Dataset GSE101123, a dataset profiled circRNA expression in BC tissue and mammary gland tissue, was obtained from GEO database. Then the raw data were analyzed with R studio to screen differentially expressed circRNAs in BC. According to the criteria of | log_2_(fold change) | > 2 and *P* < 0.05, a total of 7 depleted circRNAs and 12 enriched circRNAs were identified (Fig. 1a). The expression of circRPPH1 (i.e., hsa_circRNA_001846) was significantly higher in BC tissue than in mammary gland tissue (Fig. 1b). CircRPPH1, which located on chr14:20811436-20811559 according to the UCSC Genome Browser (http://genome.ucsc.edu/), was a 123bp-long circRNA formed by backsplicing of RPPH1 exon 1 (Fig. 1c). The total RNAs of MDA-MB-231 and MCF-7 cells were treated with RNase R exonuclease and the results showed that circRPPH1 could resist the digestion while the linear RPPH1 was degraded (Fig. 1d, e). It indicated that circRPPH1 had the circular structure. In addition, RT-PCR were conducted with both convergent primers and divergent primers. The circular property was confirmed according to the result of agarose gel electrophoresis (Fig. 1f).

As for the subcellular distribution of circRPPH1, FISH assay was employed in MDA-MB-231 cells and MCF-7 cells to preliminarily investigate the location of circRPPH1. The results indicated that circRPPH1 was mainly stained in cytoplasm of BC cells (Fig. 1g). The subcellular fractionation employing GAPDH as cytoplasmic control transcripts and U6 as nuclear control transcripts also indicated that about 75% circRPPH1 were in the cytoplasm of BC cells (Fig. 1h, i).

### Highly expressed circRPPH1 lead to BC progression in vitro

**Fig. 2:**
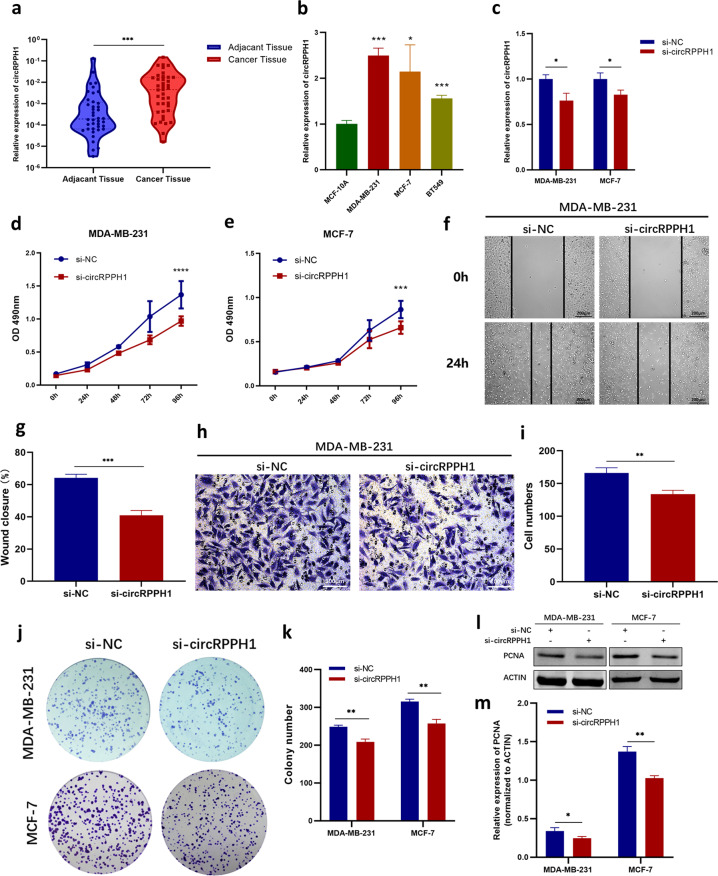
CircRPPH1 was enriched in BC and knockdown of circRPPH1 inhibited the growth of BC in vitro. **a** The expression of circRPPH1 was detected in BC tissues and their paired adjacent tissues (*N* = 40). **b** The expression of circRPPH1 in BC cells (MDA-MB-231, MCF-7, and BT549) was compared to that in mammary epithelial cells, MCF-10A. **c** CircRPPH1 levels were detected after transfecting BC cells with si-NC or si-circRPPH1. **d**–**e** Effect of si-circRPPH1 on the proliferation of MDA-MB-231 (**d**) and MCF-7 (**e**) cells were investigated by MTT assay. **f**–**g** The migration of MDA-MB-231 cells was analyzed by wound-healing assay. Photographs were taken at 0 h and 24 h after scratching (**f**). Wound closure was analyzed (**g**). **h**–**i** Transwell assay was conducted to analyze the migration of MDA-MB-231 cells. Photographs were taken at 20 h after seeding (**h**). Cell numbers were counted (**i**). **j**–**k** Effect of si-circRPPH1 on colony formation ability of BC cells (**j**). The number of cell colonies were counted (**k**). **l**–**m** The levels of PCNA protein were analyzed by western blotting. ACTIN was employed as internal control. *P < 0.05, **P < 0.01, ***P < 0.001, ****P < 0.0001.

To explore the expression of circRPPH1 in BC tissues and adjacent tissues, we conducted RT-qPCR in 40 paired tissues from BC patients. The results indicated that the expression of circRPPH1 in BC tissues was significantly higher than in adjacent tissues (Fig. 2a). In addition, circRPPH1 expression was elevated in BC cell lines (MDA-MB-231, MCF-7, and BT549) compared to normal breast epithelial cell line MCF-10A (Fig. 2b). These hinted that circRPPH1 might play a role in promoting BC progression.

**Fig. 3:**
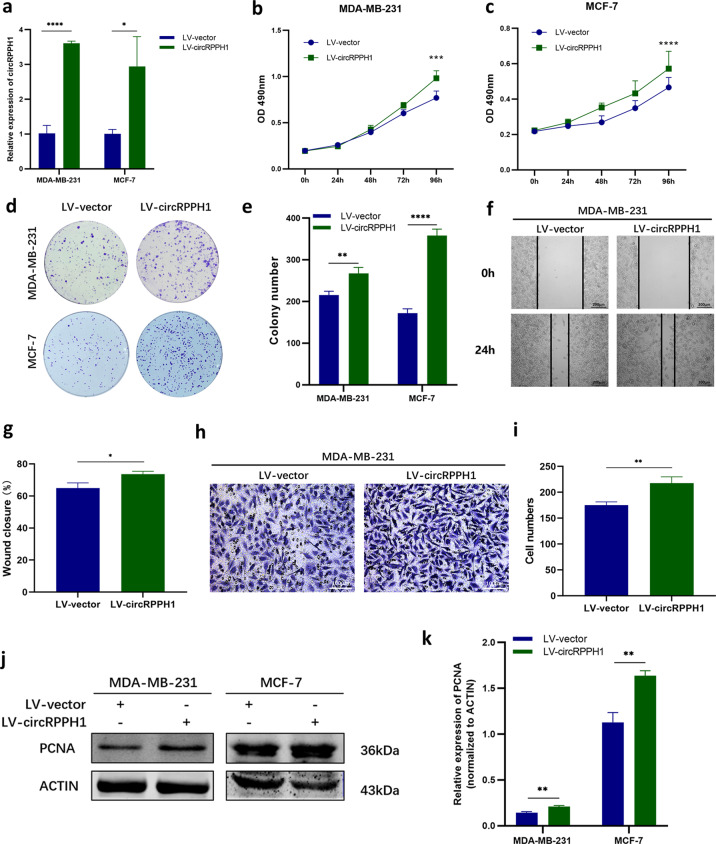
Overexpression of circRPPH1 promoted BC proliferation and migration in vitro. **a** Efficiency of LV-circRPPH1 was determined by RT-qPCR. **b**–**c** MTT assay was used to compare the proliferation of LV-vector group and LV-circRPPH1 group in MDA-MB-231 (**b**) and MCF-7 (**c**) cells. **d**–**e** Colony formation assay was conducted to investigate the colony formation ability (**d**). The number of colonies were counted (**e**). **f**–**g** The migration of MDA-MB-231 cells transfected with LV-vector or LV-circRPPH1 was analyzed by wound-healing assay. Photographs were taken at 0 h and 24 h after scratching (**f**). Wound closure was analyzed (**g**). **h**–**i** Transwell assay was conducted to analyze the migration of MDA-MB-231 cells after transfecting with LV-vector or LV-circRPPH1. Photographs were taken at 20 h after seeding (**h**) and cell numbers were counted (**i**). **j**–**k** The protein levels of PCNA in LV-vector group and LV-circRPPH1 group were analyzed by western blotting. ACTIN was employed as internal control. *P < 0.05, **P < 0.01, *** *P* < 0.001, ****P < 0.0001.

The biological functions of circRPPH1 in BC were investigated in vitro. We first transfected BC cells with specific siRNA of circRPPH1 (si-circRPPH1 and circRPPH1 si-2), and si-NC served as a negative control. The level of circRPPH1 was indeed knocked down by si-circRPPH1 (Fig. 2c) and circRPPH1 si-2 (Fig. S1a). MTT assay showed that the proliferation of BC cells was decreased when circRPPH1 was inhibited (Figs. 2d, e, S1b, S1c). The migration of MDA-MB-231 cells was limited by circRPPH1 inhibition (Figs. 2f–i, S1f–S1i). In addition, the colony formation ability was also reduced after circRPPH1 knockdown (Figs. 2j, k, S1d, S1e). The expression of PCNA, a protein reflecting cell proliferation, was reduced in the si-circRPPH1 group compared to si-NC group (Fig. 2l, m). To further validate the tumor-promoting function of circRPPH1 in BC, we constructed BC cells that could stably express circRPPH1 at high levels (LV-circRPPH1) compared to the control group (LV-vector) (Fig. 3a). The MTT assay, wound-healing assay, transwell assay and colony formation assay demonstrated that the proliferation, migration, and colony formation ability were promoted after elevating the expression of circRPPH1 (Fig. 3b–i). The PCNA level also increased in LV-circRPPH1 group, which further supported that circRPPH1 lead to BC progression in vitro (Fig. 3j, k). Furthermore, the relationships between circRPPH1 levels and clinical characteristics of 40 BC patients were also analyzed. The level of circRPPH1 was associated with age and molecular subtypes but there were no significant associations with stage, lymph node status, recurrence, and metastasis (Table 1).

**Table 1:** The relationships between the expression of circRPPH1 and clinical characteristics in BC patients.

Clinical characteristics	Total	circRPPH1 level		*P* value
		High (*n* = 29)	Low (*n* = 11)	
Age at diagnosis				
<60	18	10	8	0.040*
≥60	22	19	3	
Molecular subtype				
Luminal A	12	12	0	0.018*
Luminal B	19	12	7	
HER2-positive	5	4	1	
TNBC	4	1	3	
Stage				
I and II	36	25	11	0.560
III and IV	4	4	0	
Lymph node status				
Negative	35	24	11	0.298
Positive	5	5	0	
Recurrence and metastasis				
No	38	27	11	1.000
Yes	2	2	0	

*P < 0.05.

Bold values indicate statistical significance.

### CircRPPH1 acted as a miRNA sponge of miR-512-5p

**Fig. 4:**
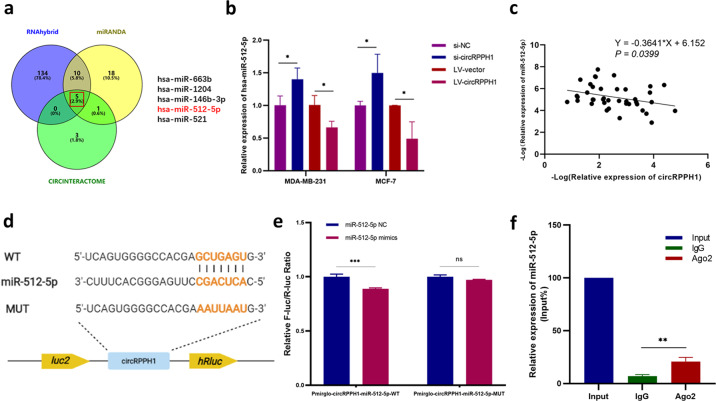
CircRPPH1 acted as a miR-512-5p sponge. **a** Potential target miRNAs of miR-512-5p were predicted by databases and presented in Venn diagram. **b** RT-qPCR was conducted to evaluate the expression of miR-512-5p in BC cells transfected with si-NC, si-circRPPH1, LV-vector or LV-circRPPH1. **c** The correlation between circRPPH1 and miR-512-5p in BC tissues was analyzed by RT-qPCR (*N* = 40). **d** Putative binding sites between circRPPH1 and miR-512-5p were predicted. Plasmids containing WT (up) or MUT (down) sequences of the putative binding sites were constructed. **e** Dual-luciferase reporter assay was performed to validate that circRPPH1 could directly bind to miR-512-5p. **f** The enrichment of miR-512-5p was detected after immunoprecipitation with AGO2 in RIP assay. *P < 0.05, **P < 0.01, ***P < 0.001, ****P < 0.0001.

Since the biological functions of circRNAs were closely related to their subcellular distributions, we assumed that circRPPH1 might act as a miRNA sponge in BC to exert its tumor-promoting functions according to its cytoplasmic location. The potential target miRNAs of circRPPH1 were firstly predicted via RNAhybrid, miRANDA and Circinteractome. As the Venn diagram showed, a total of 5 miRNAs (miR-663b, miR-1204, miR-146b-3p, miR-512-5p and miR-521) were predicted to act as binding partners of circRPPH1 (Fig. 4a). Investigation by RT-qPCR demonstrated that the expression of miR-512-5p elevated after knocking down circRPPH1 expression in BC cells, while miR-512-5p expression decreased after overexpressing circRPPH1 (Fig. 4b). Furthermore, miR-512-5p was negatively correlated to the circRPPH1 level in BC tissues, indicating that circRPPH1 might serve as a sponge of miR-512-5p (Fig. 4c). According to the predicted binding sites of circRPPH1 and miR-512-5p, we constructed WT and MUT plasmid of the complementary sequence and conducted dual-luciferase assay (Fig. 4d). The luciferase reporter activity suggested that circRPPH1 could bind to miR-512-5p through the predicted binding sites (Fig. 4e). Moreover, the result of RIP assay indicated that miR-512-5p existed in the form of miR-512-5p-AGO2 complex (Fig. 4f). Taken together, these results showed that circRPPH1 served as a sponge of miR-512-5p.

### MiR-512-5p served as a tumor suppressor in BC cells

**Fig. 5:**
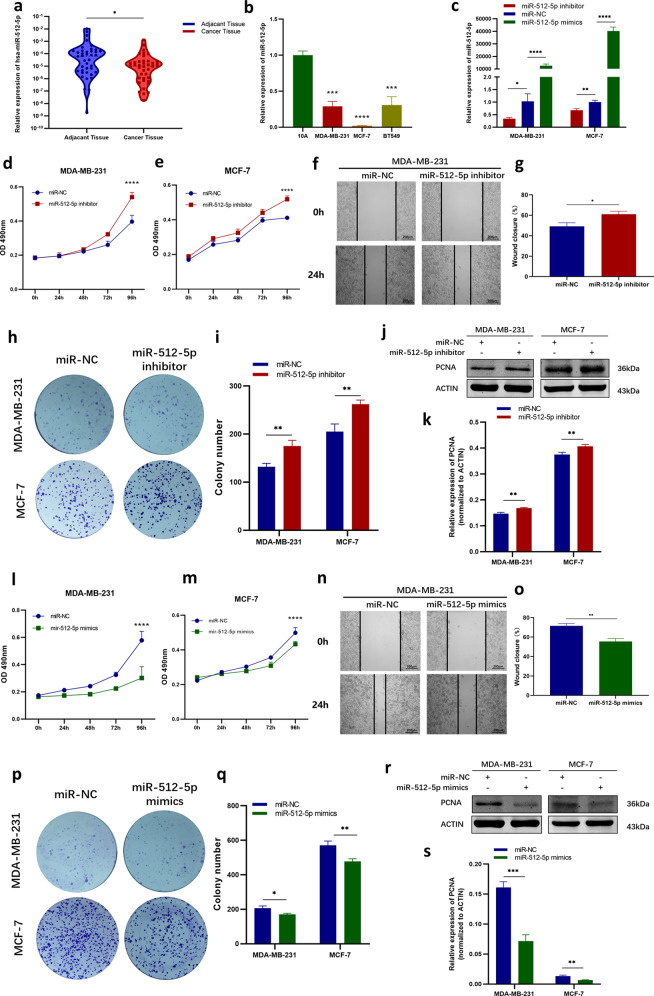
MiR-512-5p acted as a tumor suppressor in BC. **a** MiR-512-5p level was detected in BC tissues and paired adjacent tissues (*N* = 40). **b** The expression of miR-512-5p in BC cells (MDA-MB-231, MCF-7, and BT549) was compared to that in mammary epithelial cells, MCF-10A. **c** Efficiency of miR-512-5p inhibitor and mimics were determined by RT-qPCR. **d**–**e** The proliferation of MDA-MB-231 (**d**) and MCF-7 (**e**) cells was detected by MTT assay after transfecting with miR-NC or miR-512-5p inhibitor. **f**–**g** Wound-healing assay was conducted to compare the migration of MDA-MB-231 cells in miR-NC group and miR-512-5p inhibitor group. Photographs were taken at 0 h and 24 h after scratching (**f**). Wound closure was analyzed (**g**). **h**–**i** Colony formation assay was performed in miR-NC group and miR-512-5p inhibitor group in MDA-MB-231 and MCF-7 cells (**h**). The number of cell colonies were counted (**i**). **j**–**k** The protein levels of PCNA in miR-NC group and miR-512-5p inhibitor group were detected by western blotting. ACTIN was employed as internal control. **l**–**m** MTT assay was conducted to compare the proliferation of miR-NC group or miR-512-5p mimics group in MDA-MB-231 (**l**) and MCF-7 (**m**) cells. **n**–**o** The migration of MDA-MB-231 cells transfected with miR-NC or miR-512-5p mimics was analyzed by wound-healing assay. Photographs were taken at 0 and 24 h after scratching (**n**). Wound closure was analyzed (**o**). **p**–**q** Colony formation assay was performed to compare the colony formation ability of BC cells in the miR-NC group and miR-512-5p mimics group (**p**). The number of cell colonies were counted (**q**). **r**–**s** The protein level of PCNA in miR-NC group and miR-512-5p mimics group were analyzed by western blotting. The expression level was normalized to ACTIN. *P < 0.05, **P < 0.01, ***P < 0.001, ****P < 0.0001.

In order to clarify the biological function of miR-512-5p in BC, the expression of miR-512-5p in both BC tissues and cell lines were detected, and the results of RT-qPCR indicated that the levels of miR-512-5p were lower in BC tissues and cell lines (Fig. 5a, b). The inhibitor and mimics of miR-512-5p were transfected into BC cells respectively and the levels of miR-512-5p were changed correspondingly (Fig. 5c). After transfecting miR-512-5p inhibitor, the proliferation, migration and colony formation ability of BC cells were promoted (Fig. 5d–k). On the contrary, overexpressing miR-512-5p impaired the proliferation and migration of BC cells (Fig. 5l–s). These suggested that miR-512-5p functioned as a tumor suppressor in BC.

### STAT1 was a direct target gene of miR-512-5p

**Fig. 6:**
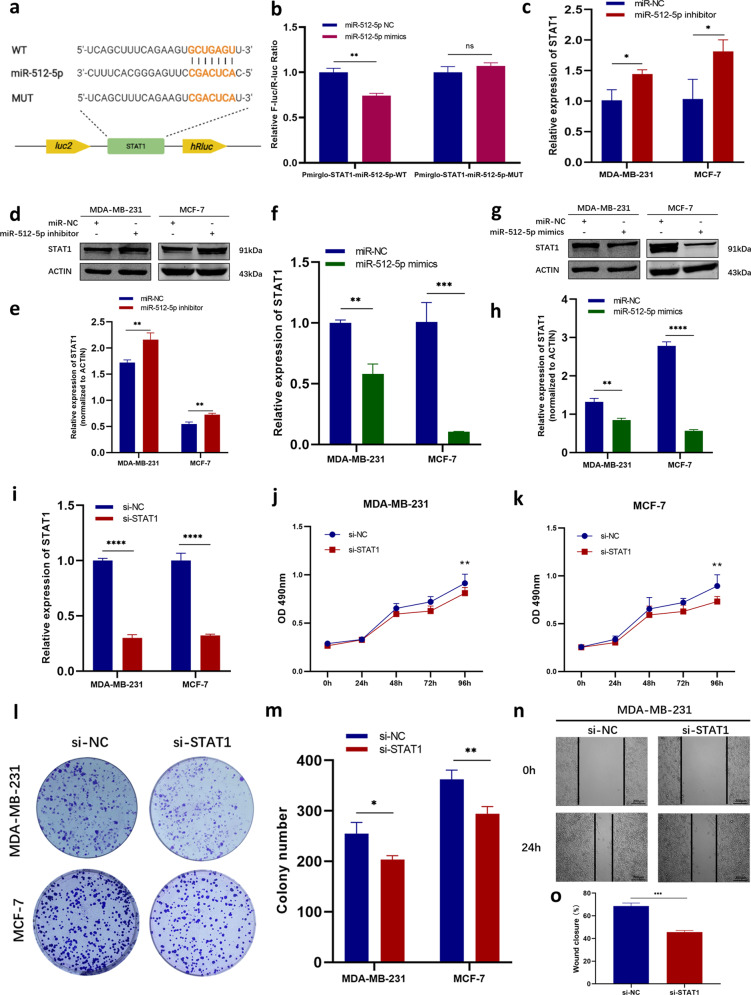
STAT1, an oncogene of BC, was directly regulated by miR-512-5p. **a** Putative binding sites between miR-512-5p and STAT1 3′-UTR were predicted. Plasmids containing WT (up) or MUT (down) sequences of the putative binding sites were constructed. **b** Dual-luciferase reporter assay was performed to detect the binding between miR-512-5p and STAT1 3′-UTR. **c** The transcription level of STAT1 in BC cells transfected with miR-NC or miR-512-5p inhibitor was detected by RT-qPCR. **d**–**e** The protein levels of STAT1 in miR-NC group and miR-512-5p inhibitor group were analyzed by western blotting. ACTIN was employed as internal control. **f** The mRNA level of STAT1 was detected by RT-qPCR after overexpression of miR-512-5p. **g**–**h** The protein levels of STAT1 in miR-NC group and miR-512-5p mimics group were analyzed by western blotting. ACTIN was used as internal control. **i** Efficiency of si-STAT1 was determined by RT-qPCR. **j**–**k** MTT assay was conducted to explore the proliferation of MDA-MB-231 (**j**) and MCF-7 (**k**) cells when STAT1 was inhibited by si-STAT1. **l**–**m** Colony formation assay was performed to compare the colony formation ability of si-NC group and si-STAT1 group (**l**). Colony numbers were counted (**m**). **n**–**o** The migration of MDA-MB-231 cells transfected with si-NC or si-STAT1 was analyzed by wound-healing assay. Photographs were taken at 0 and 24 h after scratching (**n**). Wound closure was analyzed (**o**). *P < 0.05, **P < 0.01, ***P < 0.001, ****P < 0.0001.

Based on the prediction via Targetscan, we assumed that STAT1 was one of the potential target genes of miR-512-5p (Fig. 6a). The plasmids containing WT or MUT of the 3′-UTR of STAT1 were then constructed according to the predicted binding sites (Fig. 6a). The result of the dual-luciferase assay suggested that the 3′-UTR of STAT1 was a direct target of miR-512-5p (Fig. 6b). The mRNA levels and protein levels of STAT1 were both detected after inhibition or elevation of miR-512-5p. The levels of STAT1 mRNA and protein were contrary to the change trend of miR-512-5p (Fig. 6c–h), indicating that miR-512-5p directly targeted on STAT1 mRNA. After inhibiting the expression of STAT1 (Fig. 6i), the in vitro biological function analysis showed that STAT1 was an oncogene of BC (Fig. 6j–o), which was opposite to the biological function of miR-512-5p. In short, STAT1 was a direct downstream target of miR-512-5p in BC.

### CircRPPH1 regulated BC progression via circRPPH1-miR-512-5p-STAT1 axis

**Fig. 7:**
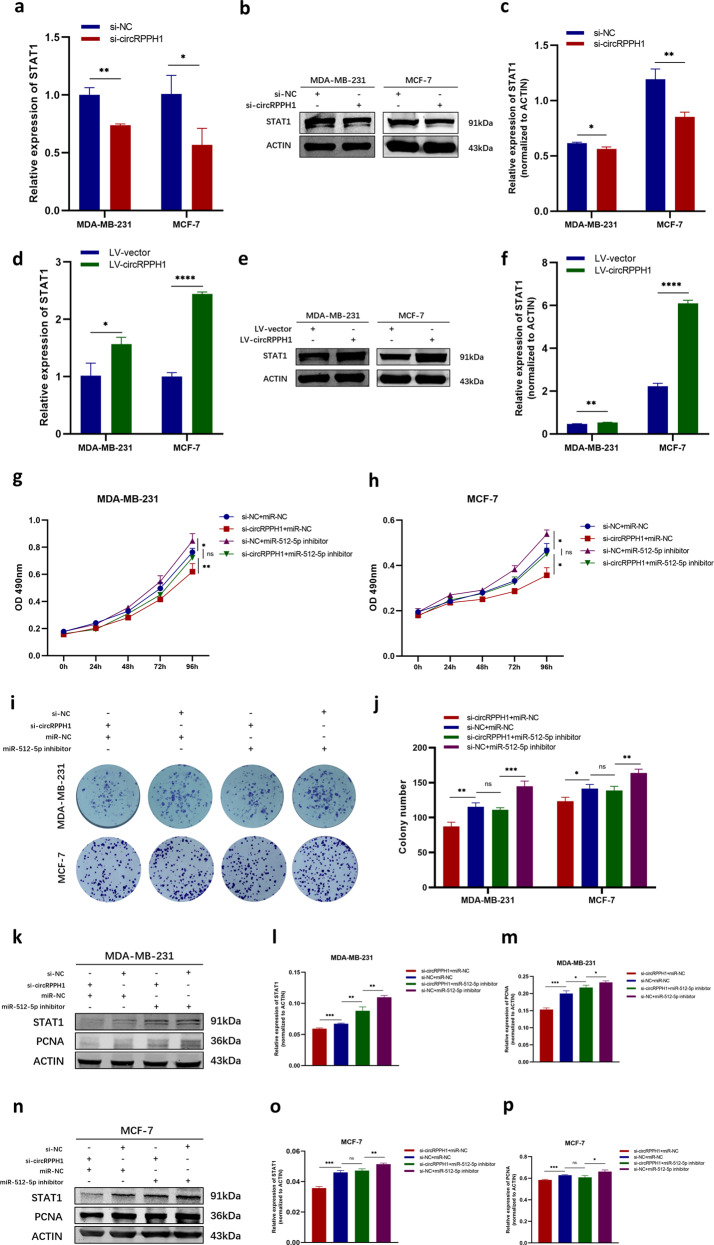
CircRPPH1 regulated STAT1 via circRPPH1-miR-512-5p-STAT1 axis. **a** Effect of si-circRPPH1 on the level of STAT1 mRNA was detected by RT-qPCR. **b**–**c** Effect of si-circRPPH1 on the protein level of STAT1. ACTIN was employed as internal control. **d** The transcription level of STAT1 was detected by RT-qPCR after overexpression of circRPPH1. **e**–**f** The protein level of STAT1 was analyzed by western blotting after transfecting BC cells with LV-vector or LV circRPPH1. The protein levels were normalized to ACTIN. **g**–**h** MTT assay was conducted to investigate whether the inhibitory effect of si-circRPPH1 on BC cell proliferation could be rescued by miR-512-5p inhibitor. **i**–**j** The effect of co-transfecting si-circRPPH1 and miR-512-5p inhibitor on colony formation was detected by colony formation assay (**i**). The cell colony numbers were counted (**j**). **k**–**p** The protein levels of STAT1 and PCNA were presented by western blotting assay in the rescue experiment in both MDA-MB-231 (**k**–**m**) and MCF-7 cells (**n**–**p**). ACTIN was taken as the internal control of protein levels. *P < 0.05, **P < 0.01, ***P < 0.001, ****P < 0.0001.

Considering the regulatory relationship between circRPPH1-miR-512-5p and miR-512-5p-STAT1, we wondered whether circRPPH1 promoted BC progression via the circRPPH1-miR-512-5p-STAT1 axis. First the mRNA levels and protein levels of STAT1 in BC cells were detected after altering circRPPH1 expression. When circRPPH1 was inhibited by si-circRPPH1, the mRNA levels and protein levels of STAT1 were both reduced in MDA-MB-231 and MCF-7 cells (Fig. 7a–c). On the contrary, overexpression of circRPPH1 upregulated the expression of mRNA and protein of STAT1 (Fig. 7d–f). To further investigate the biological role of circRPPH1-miR-512-5p-STAT1 axis in BC, rescue assays were conducted by co-transfecting si-circRPPH1 and miR-512-5p inhibitor into BC cells. Comparing to transfecting si-circRPPH1 alone, the co-transfection could partially eliminate the influence of si-circRPPH1 on the proliferation and colony formation ability of BC cells, which indicated that miR-512-5p could rescue the oncogenic function of circRPPH1 on BC (Fig. 7g–j). Consistently, downregulation of STAT1 and PCNA protein by si-circRPPH1 could be partially reversed by miR-512-5p inhibition (Fig. 7k–p). Considering the results above, we verified that circRPPH1 played its oncogenic role in BC via circRPPH1-miR-512-5p-STAT1 axis.

### CircRPPH1 played an oncogenic role in vivo

**Fig. 8:**
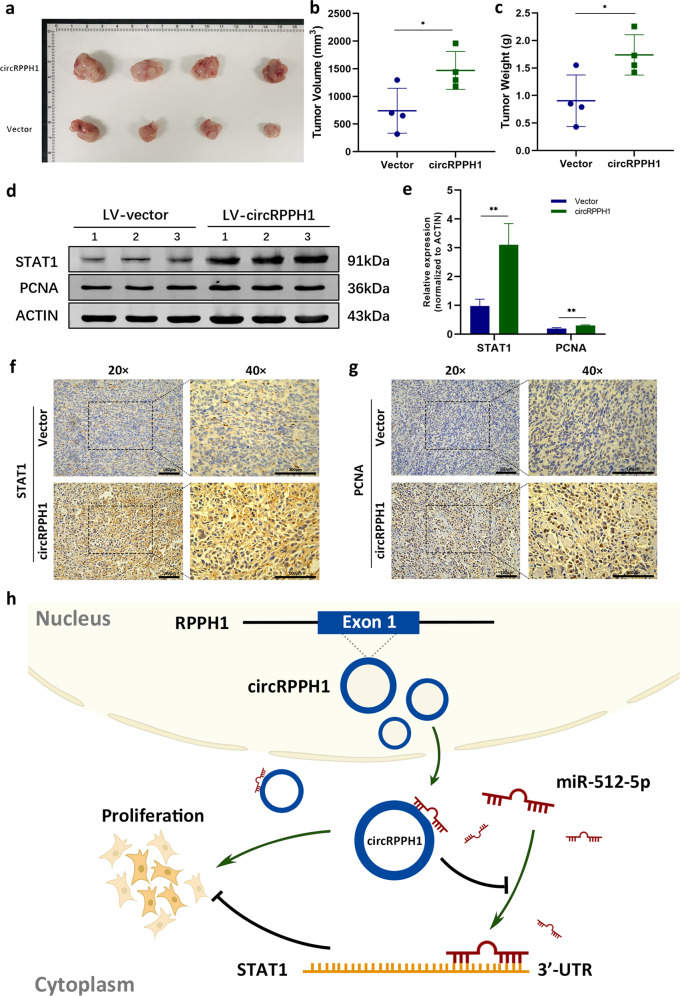
CircRPPH1 promoted tumorigenesis of BC in vivo. **a** Representative photograph of the xenografts was taken after sacrificing the BALB/c nude mice in circRPPH1 group (*N* = 4) and vector group (*N* = 4). **b**–**c** The tumor volume (**b**) and weight (**c**) of xenografts were calculated and weighted respectively. **d**–**e** Total proteins were extracted from LV-vector group and LV-circRPPH1 group of xenografts. The expression of STAT1 and PCNA in extracted proteins were analyzed by western blotting assay. **f**–**g** IHC assay was conducted to detect the expression of STAT1 (**f**) and PCNA (**g**) protein in two groups of xenografts. Representative photographs were taken when the objective lens magnifications were 20 times and 40 times. **h** A schematic diagram illustrated the proposed mechanism of circRPPH1-miR-512-5p-STAT1 axis in BC. Cytoplasmic circRPPH1 sequestered miR-512-5p from the 3′-UTR of STAT1 by sponging miR-512-5p, resulting in inhibiting the suppressive role of miR-512-5p and stabilization of STAT1 in BC. *P < 0.05, **P < 0.01, ***P < 0.001, ****P < 0.0001.

To explore the biological function of circRPPH1 in vivo, the xenograft experiment was performed in BALB/c nude female mice. MDA-MB-231 cells transfected with LV-circRPPH1 or LV-vector were injected into BALB/c nude mice respectively. The tumors dissected from sacrificed mice were photographed (Fig. 8a). Tumor volume and weight indicated that overexpression of circRPPH1 promoted the progression of BC in vivo (Fig. 8b, c). Total proteins extracted from xenografts were analyzed and the expression of STAT1 and PCNA were increased in LV-circRPPH1 group (Fig. 8d, e). Consistently, IHC analysis of xenografts also indicated that circRPPH1 promoted the expression of STAT1 and PCNA (Fig. 8f, g). These suggested that circRPPH1 acted as an oncogene in BC in vivo.

Taken together, the highly expressed circRPPH1 promoted BC progression via circRPPH1-miR-512-5p-STAT1 axis. The schematic diagram of how circRPPH1 acted as an oncogene in BC was showed in Fig. 8h.

## Discussion

Since the discovery of circRNAs, there have been great progresses in the research field of circRNAs. Numerous of circRNAs have been proved to participate in the biogenesis and progression of multiple tumors including BC [17]. Comparing to other ncRNAs, the covalently closed ring structure without 5′-caps or 3′-poly(A) tails makes circRNAs more stable and the half-time of circRNAs longer. The superiority of stability gives circRNAs more advantages to become potential biomarkers and therapeutic targets for malignant tumors [18].

By analyzing data from GEO database, we identified that circRPPH1 was highly expressed in BC, which indicated that it might play a role in BC. Consistently, the expression of circRPPH1 in 40 BC tissues and cells were also elevated. In addition, circRPPH1 level was associated with age and molecular subtypes. Paradoxically, circRPPH1 expression was relatively lower in triple-negative breast cancer (TNBC) which is the most aggressive subtype of BC. We supposed that the number of TNBC patients involved in this study was too small (*N* = 4) and it might cause bias. Furthermore, there were no significant associations between circRPPH1 levels and other clinical characteristics (tumor stage, lymph node status, recurrence and metastasis). Thus, it still required more clinical samples and longer patient follow-up to further investigate the relationship between circRPPH1 level and these clinical characteristics.

As for the biological function of circRPPH1 in BC, gain- and loss-of-function experiments in vitro suggested that circRPPH1 promoted the proliferation, colony formation and migration of BC cells. Consistently, overexpression of circRPPH1 promoted BC progression in vivo. These uncovered the oncogenic role of circRPPH1 in BC.

Previous studies have revealed multiple biological functions of circRNAs including acting as competing endogenous RNAs (ceRNAs) or sponges of miRNAs [19, 20], interacting with proteins [21–23], translating into poly-peptides [24, 25] and regulating transcription of genes [10]. Furthermore, it is widely accepted that the biological functions of circRNAs are closely related to their subcellular distribution. The most common function of cytoplasmic circRNAs is miRNA sponge [26]. MiRNA response elements enriched on circRNAs are regarded as the interacting positions between circRNAs and miRNAs and the interactions make circRNAs sponging miRNAs and therefore inhibiting the function of miRNAs on target genes [27]. Here it was suggested that circRPPH1 was mainly located in the cytoplasm by FISH assay and subcellular fraction. After predicting the potential downstream miRNAs, the RIP assay and dual-luciferase reporter assay demonstrated that circRPPH1 directly binding to miR-512-5p. In vitro experiments indicated that miR-512-5p played an inhibitory role in the progression of BC. The negative correlation between the levels of circRPPH1 and miR-512-5p also supported that circRPPH1 was a miRNA sponge of miR-512-5p.

To further identify the downstream of miR-512-5p, the potential target genes of miR-512-5p were predicted. MiRNAs mainly combine to the 3′-UTR of mRNAs and form RNA-induced silencing complex to inducing the degradation or silencing of their target genes [28]. The interaction between miR-512-5p and STAT1 3′-UTR was proved by dual-luciferase reporter assay, which clarified that STAT1 was a direct target gene of miR-512-5p. Previous studies indicated that STAT1 was a double-edged sword in tumor progression [29]. Here the biological functions of STAT1 were explored and verified that STAT1 was an oncogene in BC. In addition, it was noticed that the mRNA and protein levels of STAT1 were positively correlated to the level of circRPPH1. Therefore, we performed rescue experiments and verified that circRPPH1 affecting STAT1 by sponging miR-512-5p.

Taken together, we identified a highly expressed circRNA, circRPPH1, played an oncogenic role in BC via circRPPH1-miR-512-5p-STAT1 axis, which might provide a novel biomarker and potential therapeutic target of BC.

## Materials and methods

### Bioinformatic analysis

Dataset associated with circRNA expression in BC (GSE101123) was obtain from Gene Expression Omnibus (GEO, https://www.ncbi.nlm.nih.gov/geo/) database. Differential expression analysis was conducted by R studio and the results were showed in volcano plot and box plot.

### Clinical samples and information

Tumor tissues and adjacent tissues from BC patients (*N* = 40) were collected in the Department of Breast and Thyroid, Shanghai Tenth People’s Hospital (Shanghai, China) and all tissue samples were stored in liquid nitrogen immediately after resection. All patients were newly diagnosed BC patients without any interference before surgical removal of tumor. All patients had given their consents and the clinical information of all patients were available.

### Cell culture and transfection

Normal breast epithelial cell line (MCF-10A) and BC cell lines (MDA-MB-231 and MCF-7) were acquired from the Chinese Academy of Sciences (Shanghai, China). All cells were cultured in a 5% CO_2_ incubator at 37 °C. The medium for MCF-10A cells was Mammary Epithelial Basal Medium (MEBM) (Cambrex, USA). The BC cell lines were cultured in Dulbecco’s Modified Eagle’s Medium (DMEM, Gibco, USA) with 10% Fetal Bovine Serum (FBS, Gibco, USA) and Penicillin-Streptomycin Liquid (penicillin, 100 units/ml and streptomycin, 100 μg/ml, Enpromise, China). Lipofectamine® 3000 (Invitrogen, USA) was used for transient transfection following the manufacturer’s instructions. Small interfering RNAs targeting on circRPPH1 (si-circRPPH1 and circRPPH1 si-2) and their negative control (si-NC) were purchased from IBSbio (Shanghai, China). The sequences were as follows: si-circPRRH1: Sense 5′-CGGGGAGGGAAGCUCAUCATT-3′, anti-sense 5′-UGAUGAGCUUCCCUCCCCGTT-3′; circRPPH1 si-2: Sense 5′-AGCUUCGGGGAGGGAAGCUTT-3′, anti-sense 5′-AGCUUCCCUCCCCGAAGCUTT-3′; si-NC: Sense 5′-UUCUCCGAACGUGUCACGUTT-3′, anti-sense 5′-ACGUGACACGUUCGGAGAATT-3′. Inhibitors, mimics and negative control (miR-NC) of miR-512-5p were synthesized by RiboBio (Guangzhou, China). Lentiviral plasmid for overexpressing circRPPH1 and vector were designed by HarO Life (Shanghai, China) and transfected with agents from ZorinBio (Shanghai, China).

### RNase R treatment

RNAs extracted from MDA-MB-231 and MCF-7 cells were treated with Ribonuclease R (RNase R) at 37 °C for 30 min under standard assay conditions. After enzyme inactivation, the processed RNAs were detected by RT-qPCR.

### Fluorescent in situ hybridization

Specific probe of circRPPH1 for fluorescent in situ hybridization (FISH) was designed and synthesized by GenePharma (Shanghai, China). Ribo™ Fluorescent In Situ Hybridization Kit (RiboBio, Guangzhou, China) was used to detect the localization of circRPPH1. Nucleus was stained by 4′,6-Diamidino-2-Phenylindole (DAPI). Fluorescence microscope (Leica, Germany) was applied in image acquisition.

### Subcellular fraction

The Ambion® PARIS™ Kit (Invitrogen, USA) was used for subcellular fractions according to the manufacturer’s instructions. Briefly, after the preparation of whole-cell lysates, the cell components were separated into nuclear and cytoplasmic cell partitions. Nuclear and cytoplasmic RNA were then extracted respectively.

### RNA extraction, RT-PCR, and RT-qPCR

Total RNA of tissues and cells were extracted by TRIzol reagent (Invitrogen, USA). The concentration and pureness of total RNA were determined by Nanodrop 2000 spectrophotometer (Thermo Fisher, USA). To obtain cDNA, total RNA underwent reverse transcription (RT) by HiScript® III RT SuperMix for qPCR (+gDNA wiper) (Vazyme, China). Polymerase chain reaction (PCR) was performed with 2×Hieff® Robust PCR Master Mix (YEASEN, China) and the products were detected by agarose gel electrophoresis. Quantitive real-time polymerase chain reaction (RT-qPCR) was conducted with Hieff® qPCR SYBR Green Master Mix (YEASEN, China). 18S, U6 and GAPDH were employed as the internal control of circRNA, miRNA and mRNA, respectively. Sequences of primers were as follows: circRPPH1 Forward: 5′-TTGGGAAGGTCTGAGACTAGGG-3′ and Reverse: 5′-CCACTGATGAGCTTCCCTCC-3′; RPPH1 Forward: 5′-CGAGCTGAGTGCGTCCTGTC-3′ and Reverse: 5′-TCGCTGGCCGTGAGTCTGT-3′; STAT1 Forward: 5′-ATCAGGCTCAGTCGGGGAATA-3′ and Reverse: 5′-TGGTCTCGTGTTCTCTGTTCT-3′.

### MTT assay

BC cells were seeded into 96-well plates at a density of 2000 cells per well with 200 μl medium. Cell viability was detected at 0, 24, 48, 72, and 96 h after seeding, respectively. 20 μl MTT reagent (YEASEN, China) was added into each well and incubated in incubator for 4 h. The supernatant was replaced by 150 μl DMSO (Sangon, China). Optical density (OD) at 490 nm was detected by a microplate spectrophotometer (BioTek, German).

### Colony formation assay

BC cells were resuspended sufficiently in the medium to get single-cell suspension and then seeded into six-well plates at a density of 800 cells per well. The plates were incubated for about 10 days until the colonies were visible. After washing twice with phosphate-buffered saline (PBS), cell colonies were fixed with 95% ethanol for 10 min and stained with 0.1% crystalline violet. The representative photographs were taken and the number of colonies were counted.

### Wound-healing assay

Transfected MDA-MB-231 cells were seeded into six-well plates. When the cells reached 95% confluent, the 200 μl tips were held perpendicularly to the plate in order to make scratches on the surface of the cell monolayers. After gently washing cells with PBS twice, the medium was changed to DMEM medium with 2% FBS. The healing of the wound at the same position was observed under microscope and photographed at 0 and 24 h.

### Transwell assay

Transfected MDA-MB-231 cells were seeded into the upper chamber of Transwell (Corning, USA) at a density of 3 × 10^4^ cells/well. DMEM medium with 10% FBS was added into the lower chamber while DMEM medium without FBS was added into the upper chamber. After incubation in 37 °C for 20 h, the migrated cells were fixed with 4% paraformaldehyde and dyed with 0.1% crystalline violet. Representative images were photographed under microscope.

### Dual-luciferase reporter assay

To validate that miR-512-5p directly binds to circRPPH1 and targets on STAT1 3′-UTR, wild-type (WT) and mutant (MUT) reporter plasmids of both circRPPH1 and STAT1 3′-UTR were constructed according to the sequence of binding sites predicted on Circinteractome (https://circinteractome.nia.nih.gov/) and TargetScan (http://www.targetscan.org/). MiR-512-5p mimics or miR-512-5p NC was co-transfected with WT or MUT reporter plasmids into pre-seeded HEK-293T cells. Dual-luciferase reporter assay kit (YEASEN, China) was employed to detect the luciferase activities. The ratio of firefly to renilla luciferase was then calculated.

### RNA immunoprecipitation assay

RNA-Binding Protein Immunoprecipitation Kit (BersinBio, China) was used for conducting RNA immunoprecipitation (RIP) assay in MDA-MB-231 cells overexpressing circRPPH1 according to the manufacturer’s instructions. Anti-Ago2 (Abclonal, China) and Anti-IgG (Abclonal, China) were utilized for immunoprecipitation. The obtained RNAs were further analyzed by RT-qPCR.

### Protein extraction and western blotting analysis

BC cells were collected after transfection. To get protein lysates, RIPA lysis buffer (Beyotime, China) together with 1 M PMSF (Beyotime, China) were added into collected cells. The concentration of total protein was determined by BCA protein assay kit (Beyotime, China). After separation by 10% sodium dodecyl sulfate-polyacrylamide gels, proteins were transferred to nitrocellulose membranes (Beyotime, China). The membranes were blocked with 5% non-fatty milk for 1 h at room temperature and then immunoblotted with primary antibodies at 4 °C overnight. Blots were washed with PBST (PBS together with 0.1% Tween 20) and incubated in secondary antibodies at room temperature for 1 h. After washing the membranes with PBST, the bands were detected by an Odyssey Infrared scanning system (LI-COR Biosciences, USA) and then analyzed with Image Studio. The primary antibodies and their dilutions were as follows: anti-PCNA (1:1000, Abclonal, China), anti-STAT1 (1:500, Wanleibio, China) and anti-ACTIN (1:10,000, Abclonal, China).

### Xenografts experiment

BALB/c nude mice (4-week old, female) were ordered from SLAC (Shanghai, China) and divided into two groups randomly (*N* = 4 in each group). 1 × 10^6^ MDA-MB-231 cells which stably expressed vector or circRPPH1 were injected into the second mammary fat pad of mice in two groups, respectively. After 6 weeks’ observation, all mice were sacrificed by cervical dislocation and dissected to collect tumors. The tumor volume were calculated as follows: Volume (mm^3^) = width(mm)^2^ × length(mm)/2. The animal experiments were approved by Animal Care and Use Committee of Shanghai Tenth People’s Hospital.

### Immunohistochemistry

Tumor tissue from the BALB/c nude mice were fixed in 4% paraformaldehyde. The fixed tissue were then dehydrated by ethanol solution, embedded in paraffin, and sectioned into 4 μm-thick slides. After deparaffinizing, rehydrating, antigen retrieving and endogenous peroxidase blocking, the slides were incubated with antibodies (dilution 1:500) to examine the expression of PCNA and STAT1. Images were photographed by Leica Microsystems (Germany).

### Statistical analysis

Data were obtained from at least three independent experiments. The significances of differences were evaluated and presented by GraphPad Prism (v8.3.0, USA). The relationships between circRPPH1 levels and clinical characteristics were analyzed by chi-square test. Comparisons between paired specimens were analyzed by Wilcoxon matched-pairs signed rank test while unpaired Student’s *t*-test was used for unpaired samples. Two-way ANOVA was used for the analysis of MTT assay. The correlation between circRPPH1 and miR-512-5p was analyzed by simple linear regression. Data were presented as mean ± standard deviation (SD) and they were considered significant when *P*-values < 0.05.

## Supplementary information

Figure S1. Knockdown of circRPPH1 by circRPPH1 si-2 inhibited the growth of BC in vitro.

Supplementary information

Author contribution statement

## Data Availability

All data and materials are available under request by contacting with the corresponding author.

## References

[1] Sung H, Ferlay J, Siegel RL, Laversanne M, Soerjomataram I, Jemal A (2021). Global Cancer Statistics 2020: GLOBOCAN estimates of incidence and mortality worldwide for 36 cancers in 185 countries. CA Cancer J. Clin..

[2] Liang Y, Zhang H, Song X, Yang Q (2020). Metastatic heterogeneity of breast cancer: molecular mechanism and potential therapeutic targets. Semin Cancer Biol..

[3] Lei S, Zheng R, Zhang S, Wang S, Chen R, Sun K (2021). Global patterns of breast cancer incidence and mortality: a population-based cancer registry data analysis from 2000 to 2020. Cancer Commun.

[4] McDonald ES, Clark AS, Tchou J, Zhang P, Freedman GM (2016). Clinical diagnosis and management of breast cancer. J. Nucl. Med.

[5] Li J, Sun D, Pu W, Wang J, Peng Y (2020). Circular RNAs in cancer: biogenesis, function, and clinical significance. Trends Cancer.

[6] Kristensen LS, Andersen MS, Stagsted LVW, Ebbesen KK, Hansen TB, Kjems J (2019). The biogenesis, biology and characterization of circular RNAs. Nat Rev Genet.

[7] Yang Y, Gao X, Zhang M, Yan S, Sun C, Xiao F (2018). Novel role of FBXW7 circular RNA in repressing glioma tumorigenesis. J. Natl Cancer Inst..

[8] Zhang N, Nan A, Chen L, Li X, Jia Y, Qiu M (2020). Circular RNA circSATB2 promotes progression of non-small cell lung cancer cells. Mol Cancer.

[9] Chen RX, Chen X, Xia LP, Zhang JX, Pan ZZ, Ma XD (2019). N(6)-methyladenosine modification of circNSUN2 facilitates cytoplasmic export and stabilizes HMGA2 to promote colorectal liver metastasis. Nat Commun..

[10] Li Z, Huang C, Bao C, Chen L, Lin M, Wang X (2015). Exon-intron circular RNAs regulate transcription in the nucleus. Nat Struct Mol Biol..

[11] Vo JN, Cieslik M, Zhang Y, Shukla S, Xiao L, Zhang Y (2019). The Landscape of circular RNA in cancer. Cell.

[12] Verduci L, Tarcitano E, Strano S, Yarden Y, Blandino G (2021). CircRNAs: role in human diseases and potential use as biomarkers. Cell Death Dis..

[13] Verhoeven Y, Tilborghs S, Jacobs J, De Waele J, Quatannens D, Deben C (2020). The potential and controversy of targeting STAT family members in cancer. Semin Cancer Biol..

[14] Zhang Y, Liu Z (2017). STAT1 in cancer: friend or foe?. Discov Med.

[15] Huang P, Liao R, Chen X, Wu X, Li X, Wang Y (2020). Nuclear translocation of PLSCR1 activates STAT1 signaling in basal-like breast cancer. Theranostics.

[16] Hou Y, Li X, Li Q, Xu J, Yang H, Xue M (2018). STAT1 facilitates oestrogen receptor α transcription and stimulates breast cancer cell proliferation. J Cell Mol Med.

[17] Zhang HD, Jiang LH, Sun DW, Hou JC, Ji ZL (2018). CircRNA: a novel type of biomarker for cancer. Breast Cancer.

[18] Meng S, Zhou H, Feng Z, Xu Z, Tang Y, Li P (2017). CircRNA: functions and properties of a novel potential biomarker for cancer. Mol Cancer.

[19] Liu Z, Yu Y, Huang Z, Kong Y, Hu X, Xiao W (2019). CircRNA-5692 inhibits the progression of hepatocellular carcinoma by sponging miR-328-5p to enhance DAB2IP expression. Cell Death Dis..

[20] Zhou ZB, Huang GX, Fu Q, Han B, Lu JJ, Chen AM (2019). circRNA.33186 contributes to the pathogenesis of osteoarthritis by sponging miR-127-5p. Mol Ther..

[21] Zhu YJ, Zheng B, Luo GJ, Ma XK, Lu XY, Lin XM (2019). Circular RNAs negatively regulate cancer stem cells by physically binding FMRP against CCAR1 complex in hepatocellular carcinoma. Theranostics.

[22] Sun YM, Wang WT, Zeng ZC, Chen TQ, Han C, Pan Q (2019). circMYBL2, a circRNA from MYBL2, regulates FLT3 translation by recruiting PTBP1 to promote FLT3-ITD AML progression. Blood.

[23] Zhao Q, Liu J, Deng H, Ma R, Liao JY, Liang H (2020). Targeting mitochondria-located circRNA SCAR alleviates NASH via reducing mROS output. Cell.

[24] Yang Y, Fan X, Mao M, Song X, Wu P, Zhang Y (2017). Extensive translation of circular RNAs driven by N(6)-methyladenosine. Cell Res.

[25] Zhang M, Zhao K, Xu X, Yang Y, Yan S, Wei P (2018). A peptide encoded by circular form of LINC-PINT suppresses oncogenic transcriptional elongation in glioblastoma. Nat Commun..

[26] Tang X, Ren H, Guo M, Qian J, Yang Y, Gu C (2021). Review on circular RNAs and new insights into their roles in cancer. Comput Struct Biotechnol J..

[27] Li D, Zhang J, Li J (2020). Role of miRNA sponges in hepatocellular carcinoma. Clin Chim. Acta.

[28] Stavast CJ, Erkeland SJ (2019). The non-canonical aspects of microRNAs: many roads to gene regulation. Cells.

[29] Li X, Wang F, Xu X, Zhang J, Xu G (2021). The dual role of STAT1 in ovarian cancer: insight into molecular mechanisms and application potentials. Front Cell Dev Biol..

