# Supplementary figures and images for: Circular RNA circRPPH1 promotes breast cancer progression via circRPPH1-miR-512-5p-STAT1 axis
Source: Cell Death Discov. 2021 Dec 6;7:376. doi: 10.1038/s41420-021-00771-y (PMC8648777; doi:10.1038/s41420-021-00771-y)

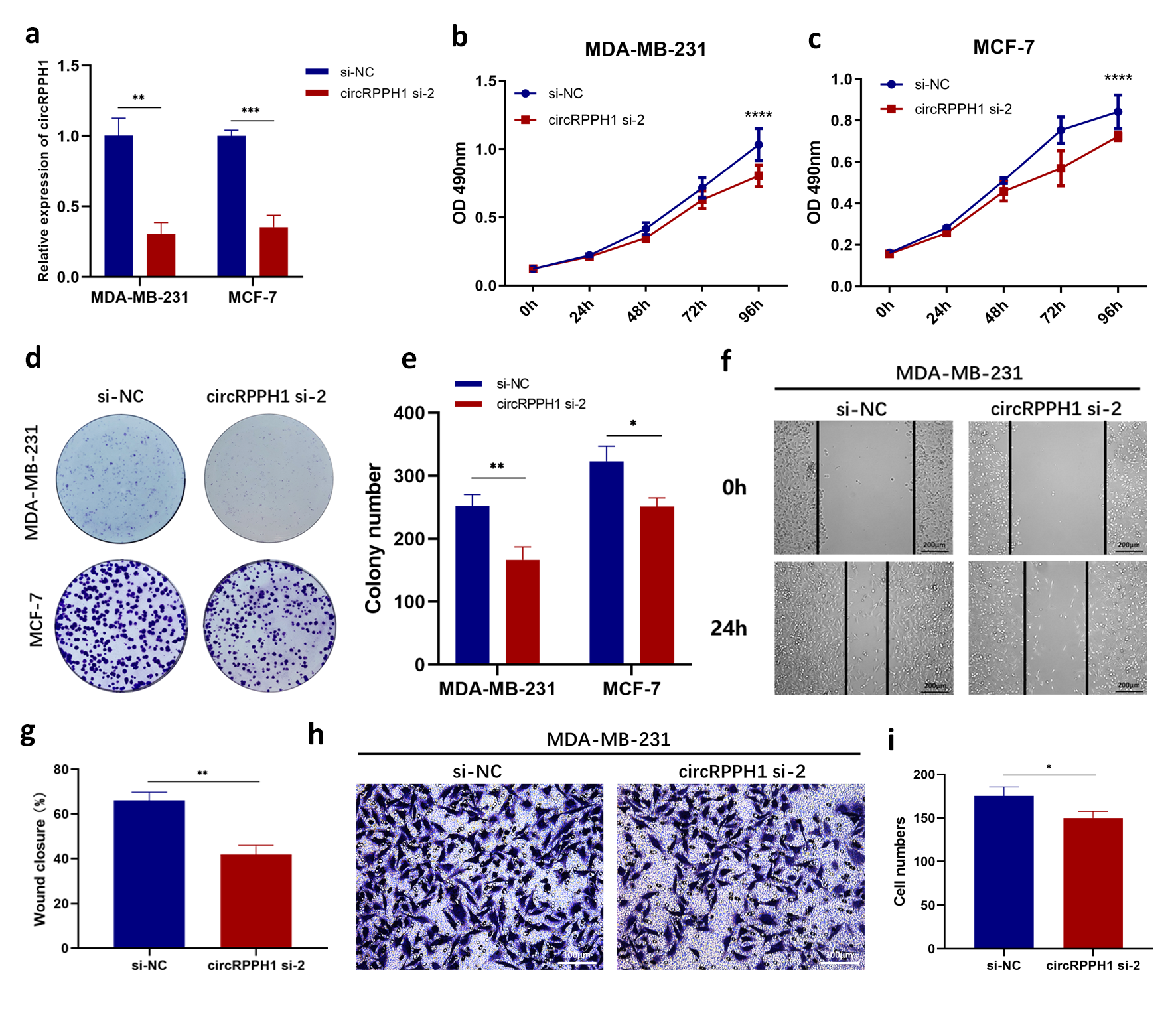

Supplement: Supplementary file 1 — Figure S1. Knockdown of circRPPH1 by circRPPH1 si-2 inhibited the growth of BC in vitro. [file 41420_2021_771_MOESM1_ESM.tif]
