# Supplementary material for: Circular RNA circRPPH1 promotes breast cancer progression via circRPPH1-miR-512-5p-STAT1 axis
Source: Cell Death Discov. 2021 Dec 6;7:376. doi: 10.1038/s41420-021-00771-y (PMC8648777; doi:10.1038/s41420-021-00771-y)
Supplement: Supplementary file 2 — Supplementary information [file 41420_2021_771_MOESM2_ESM.docx]

**Figure S1. Knockdown of circRPPH1 by circRPPH1 si-2 inhibited the growth of BC in vitro. a.** CircRPPH1 levels were detected after transfecting BC cells with si-NC or circRPPH1 si-2. **b-c.** Effect of circRPPH1 si-2 on the proliferation of MDA-MB-231 (**b**) and MCF-7 (**c**) cells were investigated by MTT assay. **d-e.** Effect of circRPPH1 si-2 on colony formation ability of BC cells (**d**). The number of cell colonies were counted (**e**). **f-g.** The migration of MDA-MB-231 cells was analyzed by wound healing assay. Photographs were taken at 0h and 24h after scratching (**f**). Wound closure was analyzed (**g**). **h-i.** Transwell assay was conducted to analyze the migration of MDA-MB-231 cells transfected with si-NC or circRPPH1 si-2. Photographs were taken at 20h after seeding (**h**). Cell numbers were counted (**i**). **P* < 0.05, ***P* < 0.01, *** *P* < 0.001, **** *P* < 0.0001.
