# Supplementary material for: Circular RNA circRPPH1 promotes breast cancer progression via circRPPH1-miR-512-5p-STAT1 axis
Source: Cell Death Discov. 2021 Dec 6;7:376. doi: 10.1038/s41420-021-00771-y (PMC8648777; doi:10.1038/s41420-021-00771-y)
Supplement: Supplementary file 3 — Author contribution statement [file 41420_2021_771_MOESM3_ESM.docx]

**Author’s contributions**

W.Z, Y.H and L.F designed this research. W.Z, Y.H, C.J, X.W, Y.Y, X.D and X.Z collected the samples and clinical information. W.Z and Y.H conducted the experiments and analyzed the results. C.J, X.W, Y.Y and L.F provided vital comments. W.Z and Y.H wrote the manuscript. All authors have read and approved the final manuscript.
